# Editorial: Women in Biomechanics and Control of Human Movement: 2022–2023

**DOI:** 10.3389/fspor.2023.1337636

**Published:** 2023-12-12

**Authors:** Kimberley S. van Schooten, Sina David, Laura E. Diamond

**Affiliations:** ^1^ Falls, Balance and Injury Research Centre, Neuroscience Research Australia, Randwick, NSW, Australia; ^2^ School of Population Health, University of New South Wales, Kensington, NSW, Australia; ^3^ Department of Human Movement Sciences, Faculty of Behavioural and Movement Sciences, Vrije Universiteit Amsterdam, Amsterdam, Netherlands; ^4^ Griffith Centre of Biomedical and Rehabilitation Engineering, Menzies Health Institute Queensland, Griffith University, Gold Coast, QLD, Australia; ^5^ School of Health Sciences and Social Work, Griffith University, Gold Coast, QLD, Australia

**Keywords:** motor control, gender disparities in academia, rolemodels, underrepresentation in science, sponsorship

**Editorial on the Research Topic** Women in Biomechanics and Control of Human Movement: 2022–2023

## Introduction

Diversity drives creativity and innovation. STEM fields, including those of biomechanics and motor control, are perceived as male-dominant domains (1). This perception is supported by the fact that men currently make up 73% of the STEM-skilled workforce (2, 3) and 70% of professional biomechanics societies (4, 5). Successful women serving as role models can inspire and encourage traditionally underrepresented groups to pursuit STEM disciplines and remain in the field (6). This Research Topic on “Women in Biomechanics and Control of Human Movement: 2022–2023” celebrates the high-quality research performed by women in the field, with the hope of inspiring the next generation of STEM leaders.

The Research Topic showcases the diverse research by women, ranging from dual-task performance under physical exertion to factors associated with sports performance and the “attractiveness” of walking. It is important to note that most of this research was conducted during the COVID19 pandemic, which disproportionally affected parents and caregivers (7). Therefore, it is even more commendable that these eight teams have been able to contribute to this Research Topic.

## Dual tasks and walking

Hogg et al. demonstrated that the effect of a cognitive dual-task changes after exercise-induced fatigue. They found that participants had better dual-task cognitive performance during lateral stepping compared to sitting, single-legged stance and walking. Moreover, while cognitive dual-task performance remained relatively stable after a moderate-to vigorous intensity 20-min treadmill walking session during sitting, single-legged stance, and walking, there was reduced dual-task performance during lateral stepping. The findings highlight the need to consider motor task and exertion when evaluating cognitive performance.

Almaijd et al. also focussed on cognitive-dual tasks, showing that performing the dual task of texting while walking had a greater impact on speed outdoors compared to walking indoors. Furthermore, they found that the accuracy of texting did not vary between environments. The findings underscore the need for education on the attentional demands of texting in dynamic, busy environments.

Tanabe et al. investigated how women modified their walking when asked to walk as attractively as possible. They found that when asked to walk attractive, women show greater energy at the hip joint after ipsilateral foot contact, greater angular disagreement between the thoracolumbar joint and neck, and a more forwards-bended head. Women who were professional models further extended their knee more during the push-off phase and pulled their upper arms backwards. The researchers confirmed the attractiveness of the walking pattern by creating stick figures, which walking patterns were rated on attractiveness by external observers. These findings highlight the complex sociocultural aspects of gait that may influence our observational assessments.

## Sports and injury biomechanics

The remaining five articles in this Research Topic focused on sports and injury biomechanics.

Powell et al. determined the effect of breast support on knee joint biomechanics during treadmill running. Their results suggest that greater breast support through supportive sports bras resulted in greater knee joint stiffness and knee extension power, and smaller knee flexion excursion. Greater knee joint stiffness has previously been associated with better running performance and lower risk of injury, suggesting that proper breast support may be important for athletes with breasts.

Lambrich and Meuhlbauer investigated how plantar pressure changes with ball speed during longline forehand and backhand strokes in elite female tennis players. The results suggest that during forehand strokes, players increased plantar pressure in the dominant foot while decreasing it in the non-dominant foot to achieve higher post-impact ball speeds. Conversely, for backhand strokes, players decreased plantar pressure in both feet to increase ball speed. The study highlights the importance of tailored physical exercises based on foot dominance and stroke technique for enhancing performance in female tennis players.

Harrison et al. compared peak and support joint moment contributions during incline and decline running in people with anterior cruciate ligament (ACL) reconstruction and matched controls. Their results showed no significant differences in joint moment contributions between limbs or groups, indicating that those with ACL reconstruction do not exhibit alterations in joint contributions during sloped running. They showed a change in ankle and knee joint moment contributions from a 10° incline to a 10° decline, suggesting a redistribution of mechanics across lower extremities. The researchers propose that factors other than support joint moment contributions (as assessed in this study) should be explored to understand the increased risk of osteoarthritis in people with ACL reconstruction.

Cederbaum et al. explored the coordination of the vastus medialis and vastus lateralis muscles during repeated sprinting to understand a sex discrepancy in patellofemoral pain syndrome. The results showed similar muscle coordination patterns and onset delays between sexes, with an increase in time delay with repeated sprinting. They observed an earlier plateau in knee extensor muscle activation amplitude in females, while mechanical work plateaued at similar sprint repetitions between sexes. The study suggests that muscle groups other than the vasti may be responsible for females' greater risk of patellofemoral pain syndrome.

Kusafuka et al. investigated the ability of people with and without baseball experience to pitch at different target positions. Participants with baseball experience exhibited lower variability in pitch location, demonstrating enhanced control over both timing and spatial accuracy. The study highlights the complex motor control mechanisms involved in accurate throwing tasks.

## Need for sponsorship of women in biomechanics and motor control

This Research Topic serves as a testament to the remarkable contributions of women in biomechanics and motor control. The diverse studies, spanning from the effect of cognition and sociocultural nuances on walking to factors associated with elite sportsmanship and injury, showcase the breadth and depth of research undertaken by women in the field. Yet, amidst these triumphs, we cannot ignore the stark reality that women receive 30% fewer citations than men for their scientific publications, a difference persisting from 1950 to today (8). This discrepancy underlines the pressing need for active promotion of women and their research.

A powerful strategy to enhance the visibility of individuals and their research is sponsorship, where the sponsor actively advocates for the protégé. It is different from mentorship, which is about providing guidance and support, fostering skill development, and sharing experiences, in that it involves management of others' view of the protégé and their work. Please join us in supporting and promoting diversity in biomechanics and motor control by actively sponsoring the articles in this Research Topic and the researchers who contributed them.

Let this Research Topic be a catalyst for change—a reminder that our work does not end with publications but extends to actively shaping an inclusive and supportive community. Through sponsorship, mentorship, and collective efforts, we can pave the way for a future where diversity is not just celebrated but embedded in the fabric of scientific culture.
